# Dimensional Accuracy Assessment of Medical Anatomical Models Produced by Hospital-Based Fused Deposition Modeling 3D Printer

**DOI:** 10.3390/jimaging11020039

**Published:** 2025-01-30

**Authors:** Kevin Wendo, Catherine Behets, Olivier Barbier, Benoit Herman, Thomas Schubert, Benoit Raucent, Raphael Olszewski

**Affiliations:** 1 Neuro Musculo Skeletal Lab (NMSK), Institut de Recherche Expérimentale et Clinique (IREC), Université Catholique de Louvain (UCLouvain), 1200 Brussels, Belgium; olivier.barbier@saintluc.uclouvain.be (O.B.); thomas.schubert@uclouvain.be (T.S.); raphael.olszewski@saintluc.uclouvain.be (R.O.); 2 Oral and Maxillofacial Surgery Lab (OMFS Lab), NMSK, IREC, Université Catholique de Louvain (UCLouvain), 1200 Brussels, Belgium; 3 Department of Pediatrics, Cliniques Universitaires Saint-Luc, 1200 Brussels, Belgium; 4 Morphology Lab (MORF), IREC, Université Catholique de Louvain (UCLouvain), 1200 Brussels, Belgium; catherine.behets@uclouvain.be; 5 Department of Orthopedic Surgery, Cliniques Universitaires Saint-Luc, 1200 Brussels, Belgium; 6 Institute of Mechanics, Materials and Civil Engineering, Université Catholique de Louvain (UCLouvain), 1348 Louvain-La-Neuve, Belgium; benoit.herman@uclouvain.be (B.H.); benoit.raucent@uclouvain.be (B.R.); 7 Department of Oral and Maxillofacial Surgery, Cliniques Universitaires Saint-Luc, 1200 Brussels, Belgium; 8 Department of Perioperative Dentistry, L. Rydygiera Collegium Medicum, Nicolaus Copernicus University, 85-067 Bydgoszcz, Poland

**Keywords:** medical 3D printing, accuracy, validation, dimensional error, hand, cone beam computed tomography, fused deposition modeling, prosthesis, 3D printing, anatomical model

## Abstract

As 3D printing technology expands rapidly in medical disciplines, the accuracy evaluation of 3D-printed medical models is required. However, no established guidelines to assess the dimensional error of anatomical models exist. This study aims to evaluate the dimensional accuracy of medical models 3D-printed using a hospital-based Fused Deposition Modeling (FDM) 3D printer. Two dissected cadaveric right hands were marked with Titanium Kirshner wires to identify landmarks on the heads and bases of all metacarpals and proximal and middle phalanges. Both hands were scanned using a Cone Beam Computed Tomography scanner. Image post-processing and segmentation were performed on 3D Slicer software. Hand models were 3D-printed using a professional hospital-based FDM 3D printer. Manual measurements of all landmarks marked on both pairs of cadaveric and 3D-printed hands were taken by two independent observers using a digital caliper. The Mean Absolute Difference (MAD) and Mean Dimensional Error (MDE) were calculated. Our results showed an acceptable level of dimensional accuracy. The overall study’s MAD was 0.32 mm (±0.34), and its MDE was 1.03% (±0.83). These values fall within the recommended range of errors. A high level of dimensional accuracy of the 3D-printed anatomical models was achieved, suggesting their reliability and suitability for medical applications.

## 1. Introduction

Additive manufacturing (AM), commonly called “3D printing”, encompasses several sophisticated engineering processes producing three-dimensional (3D) physical objects from 3D digital images [1]. These technologies are referred to as additive, in contrast to conventional substrative manufacturing processes (e.g., CNC milling), as 3D objects are manufactured layer by layer, successively, with reduced material waste [1,2]. Moreover, AM allows the production of complex and intricate geometrical shapes, hardly achievable using traditional manufacturing techniques (e.g., Injection Molding) [3]. Accessing the full capacity of AM processes requires computational modeling through the use of Computer-Aided Design (CAD) software [1,2,4]. Indeed, these systems allow the digital creation of two- and three-dimensional object designs and the simulation of their behaviors under real-life constraints [2,4]. Digital designs and models can be generated manually de novo or can be derived from specific sources (e.g., 3D scanning, medical imaging, pictures, …) [1,2]. Recent technological advancements have led to the possibility of the automatic generation of designs (i.e., generative design) based on specific criteria and constraints (e.g., performance requirement, materials, …) [5].

AM is a rapidly expanding technology that gradually finds its place in medical care [1]. Indeed, an increasing number of healthcare facilities implement 3D printing in their clinical routine for various purposes: enhancing patient education and resident training; improving surgical planning; using 3D-printed (3DP) implants, orthoses and prostheses; and so forth [1,6]. These clinical applications are supported by a growing body of evidence suggesting that AM can effectively improve care management through a reduction in technical procedure durations and costs and the enhancement of skills and knowledge [1,7].

Despite the reported added value of medical 3D printing, it is mandatory to ensure the dimensional accuracy of 3DP anatomical models in order to provide reliable, safe and effective care [8,9]. Inaccurate 3DP replicas represent a potential risk of detrimental clinical decisions that could negatively impact patient care management [8,9,10]. However, the assessment of the dimensional accuracy of 3D-printed processes is not systematically undertaken by researchers reporting clinical cases involving 3DP anatomical models [8,9,11].

Dimensional accuracy (DA) can be impacted at every stage of 3DP part production: from imaging acquisition to image post-processing and segmentation, and to the 3D printing process itself [8,11]. Although no established and universally recognized standards to validate dimensional accuracy exist to date, guidelines and recommendations from experts and reference works in the field indicate cut-offs of 1 mm for the mean absolute difference and 2% for relative dimensional error [8,12].

Studies have reported validated DA for various AM technologies: Fused Deposition modeling (FDM), Stereolithography (SLA), Selective Laser Sintering (SLS), Material Jetting (MJ) and Binder Jetting (BJ) [8]. The most common AM processes in medical 3D printing are FDM, SLA, SLS and MJ [1]. This order proportionally follows their level of affordability, their technical complexity and their printing resolution [1,2] Nevertheless, FDM 3D printers have been proven to be highly accurate, even affordable entry-level machines, in comparison to SLA and MJ, focusing the choice of technology primarily on final model application rather than theorical printing resolution [8,11,12,13].

The accuracy of numerous 3D printers using different AM processes has been validated in multiple medical disciplines: maxillofacial surgery, cardiology, neurosurgery, orthopedics, etc. [8,14]. However, very few reports have explored the dimensional accuracy of AM for producing hand models, or other related anatomical structures, despite its usage in numerous applications: hand splints, customized implants and surgical guides, patient and physician education, presurgical planning and assistive devices [6,15,16,17,18,19,20,21,22,23,24]. For example, Brouwers et al., validated their AM processes for the production of 3D-printed anatomical models for trauma surgery planning [16]. In their study, the authors analyzed nine human specimens, including three cadaveric hands, marking three linear distances on each of these [16]. Secondly, Lebowitz et al., validated the accuracy of their methods by 3D printing carpal bones from cadaveric specimens [17].

The usual imaging data sources for 3DP models are conventional Computed Tomography (CT-scanner) and Magnetic Resonance Imaging (MRI) [8]. However, Cone Beam Computed Tomography (CBCT), initially dedicated to dental and maxillofacial indications, was reported as a valid alternative to CT-scanner or MRI to detect hand bone traumatic pathologies thanks to its low radiation and faster acquisition time while providing high spatial resolution and bone contrasts [25,26,27]. Moreover, its facilitated accessibility brings the possibility to acquire radiological images from a seated patient in comparison to CT-scanner [28]. However, to date, no study has investigated its ability to provide quality imaging data to produce accurate 3D-printed hand models for medical use.

Therefore, the purpose of this research is to validate the dimensional accuracy of a professional hospital-based FDM 3D printer for medical purposes, using CBCT technology as the primary imaging data source and hand specimens as study objects.

## 2. Materials and Methods

### 2.1. Specimens’ Preparation

Two human cadaveric upper limbs, referred to as hands A and B, from two Caucasian males aged, respectively, 88 and 99 years old, were made available by the Anatomy Department of the Catholic University of Louvain (UCLouvain) (IRB00008535, Brussels, Belgium) following local ethics committee authorization (Ref 2021-30AOU-356; approved on 13 September 2021). The right hands were completely dissected, with the removal of soft tissues in order to expose the bony elements. After removing the skin and subcutaneous adipose tissue from both the palmar face and dorsum of the hand successively, each muscle group was identified and removed. Vascular and nervous structures were also extracted. However, the ligamentous structures of the hands and distal radio-ulnar joints were left intact to maintain attachment between all bones and preserve the anthropomorphic shape of each hand. No osteoarthritis was diagnosed. The dissections were conducted using conventional tools such as scalpels and dissecting scissors, forceps and needles.

The radius and ulna were sawn at approximately 2 cm from the distal radio-ulnar joint by an experienced prosector.

Anatomical landmarks were marked using Titanium Kirschner (KT) wires (10 × 2 mm) (Newbox medical GmbH, Münster, Germany) by K.W., a medical doctor, under the supervision of C.B., a professor of anatomy. They were inserted at the following specific anatomical locations on the dorsum of both hands using a 3D-printed guide: bases and heads of all five metacarpals (MCPs), of all five proximal phalanges (PPs) and of the four middle phalanges (MPs). Therefore, a landmark was defined as a bone segment marked by two KT wires set, respectively, at its proximal and distal ends. Linear measurements were thus taken of the distance separating two KT wires of a single landmark. Hence, a total of fourteen landmarks were marked on each hand, five MCPs, five PPs and four MPs. Table 1 presents all of the landmarks’ names and definitions.

**Figure 1:**
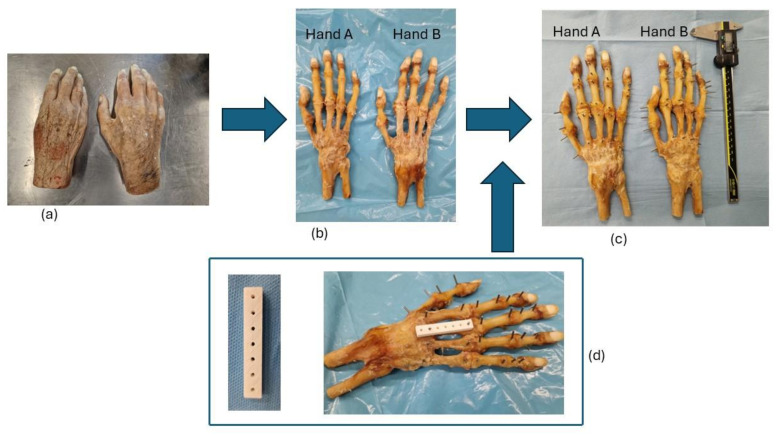
Cadaveric specimens’ preparation workflow. (**a**) Hand cadaveric specimens prior to dissection. (**b**) Dissected hands (A and B). (**c**) Dissected hand marked with Titanium landmarks. (**d**) 3D-printed guide used for inserting landmarks on bone segments.

Figure 1 illustrates the workflow to prepare the hand specimens prior to radiological image acquisition.

### 2.2. Image Acquisition

Imaging datasets of the two dissected and marked hands, named A and B, respectively, were acquired using a Cone Beam Computed Tomography (CBCT) device (Planmeca—ProMax 3D Mid, Planmeca, Finland). The radiological protocol was as follows: 90 kV, 5 mA, stereo mode, pixel size of 0.25 mm, slice thickness of 0.2 mm and field of view of 160 × 102 mm. The raw radiological data were saved and exported in Digital Imaging and Communications in Medicine (DICOM)-format files.

### 2.3. Image Processing

These DICOM data were imported to 3D Slicer v. 5.6.1 (3D Slicer software, Brigham and Women’s Hospital, Harvard Medical School, Boston, MA, USA) [29,30], an open-source segmentation software, through which the 3D reconstruction and segmentation of regions of interest (ROIs) were performed by one observer. All carpal bones were individually and manually segmented using the Paint segmenting tool and the ‘Closing’ smoothing method with a maximal 8 mm Kernel size to fill any residual inner holes. The metacarpals and phalanges were semi-manually segmented using the Threshold segmenting tool with the Otsu algorithm. The threshold range was between 450 and 3095. The ‘Closing’ smoothing algorithm was also used with a maximal 6 mm Kernel size to fill any remaining holes. Titanium landmarks, being highly dense, were automatically and optimally segmented using the threshold segmenting tool with the Shanbhag algorithm. The threshold range was between 2975 and 3095. No other smoothing method besides ‘Closing’ holes was undertaken. Artifacts were removed manually using the Erase function.

**Figure 2:**
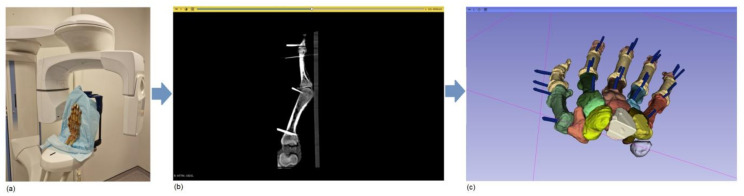
Image processing workflow. (**a**) Hand cadaveric specimen positioned in the Cone Beam Computed Tomography machine. (**b**) Radiological image of hand specimen. (**c**) Digital model of scanned hand.

The resulting digital hand models were exported in Stereolithography (STL)-format files. Figure 2 displays the workflow from image acquisition to the segmentation of ROIs.

### 2.4. Three-Dimensional Printing and Post-Processing

Each STL file was then imported into the Ideamaker software v4.3.3 (Raise3D Technologies, Irvine, CA, USA), Raise3D printer proprietary slicing software, which generated the print instruction code (G-code) for the 3D printer. The physical hand replicas were 3D-printed in a white thermoplastic polylactic acid (PLA) filament (Raise3D Technologies, Irvine, CA, USA) using a professional FDM printer, the Raise3D Pro2 plus (Raise3D Technologies, Irvine, CA, USA). This is a printer offering a large build volume (305 × 305 × 600 mm) and a dual nozzle capacity allowing dual color/material printing.

The printing parameters were as follows: layer height 0.3 mm, infill 20%, nozzle temperature 225 °C, bed temperature 60 °C, print speed 60 mm/s, addition of a raft for better adherence to the printing bed, and use of support elements. No specific post-processing was performed on the printed models other than the removal of support material. No reference sample to evaluate potential printing effects was 3D-printed using the aforementioned printing parameters prior to 3D printing the hand models.

### 2.5. Assessment of Dimensional Accuracy—Data Collection

Linear measurements were taken by two independent observers on both pairs of cadaveric and 3D-printed hand specimens using a digital Vernier caliper (Mitutoyo 150 mm, Digital Caliper, Resolution 0.01 mm, Mitutoyo, Aurora, IL, USA).

**Figure 3:**
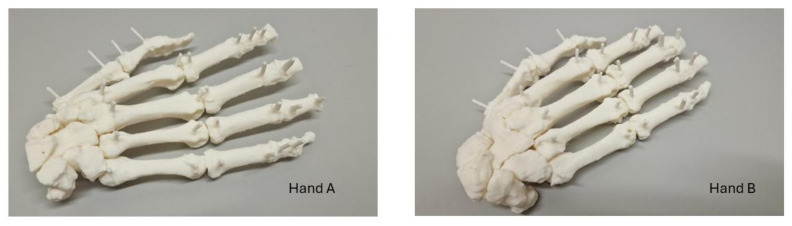
Dorsal view of 3D-printed hand models.

Each landmark distance was measured twenty times. Hence, theoretically, 280 measurements were made for each hand specimen or model. However, due to the limited exposition window of the CBCT equipment, only 10 and 13 landmarks were eventually scanned and measured in hands A and B, respectively. Figure 3 illustrates the 3D-printed hand models. Therefore, 1840 linear measurements were taken and recorded.

### 2.6. Statistical Analysis

The Absolute and Relative differences (AD; RD), or errors, were calculated for each measured linear distance based on the mean of twenty measurements. The AD was determined as the absolute difference (in mm) between the distances measured from the 3D-printed models (PMs) and the dissected hands, as illustrated in the formula below:

Absolute Difference (AD) (mm) = 3D-printed model value − Cadaveric hand value

The Relative Difference (%) was calculated by dividing the AD by the dissected hand value and multiplying by 100, as illustrated in the formula below:

Relative Difference (RD) = (|3D-printed model value − Cadaveric hand value|/Cadaveric hand value) × 100

The Mean Absolute Difference (MAD), using absolute values, and Mean Relative Difference, also named Mean Dimensional Error (MDE), were calculated for each hand–3DP model pair and for the overall study.

A paired samples *t*-test was also performed in order to compare the measurements from the dissected hands and their 3D-printed replicas. A *p*-value of 0.05 was set as statistically significant.

The statistical analysis was performed using the Microsoft Excel v2411 and IBM SPSS Statistics v29 software.

## 3. Results

Table 2 gathers information concerning the printed models. Hand B required a longer printing time and weighed slightly more than hand A due to its greater dimension and to the use of more support material as a more angulated orientation on the build plate was required. The printing time could be reduced by decreasing the infill level and limiting the support. However, as we anticipated to manipulate the models extensively, we preferred a moderate infill for robust replicas. Support material was necessary as the different angulations of the hand segments (carpal groove, metacarpals and phalanges) hindered the model from being positioned flat on the build plate.

Table 3 illustrates the correlation between the sets of measurements taken by each observer, the inter-observer agreement. To this end, the Intraclass Correlation Coefficient (ICC) was employed. Its values being between 0.998 and 1.000 for each measurement shows a high degree of agreement between the independent observers in measuring the linear distances on both hands.

Table 4 shows the intra-observer agreements of both observers. Likewise, all ICC values are comprised between 0.998 and 1.000, demonstrating an excellent agreement for each observer.

These results indicate a high degree of reproducibility, reliability and consistency among the observers.

Table 5 displays all mean measured distances for each landmark of both the cadaveric specimens and 3D-printed hand models by each observer.

**Figure A1:**
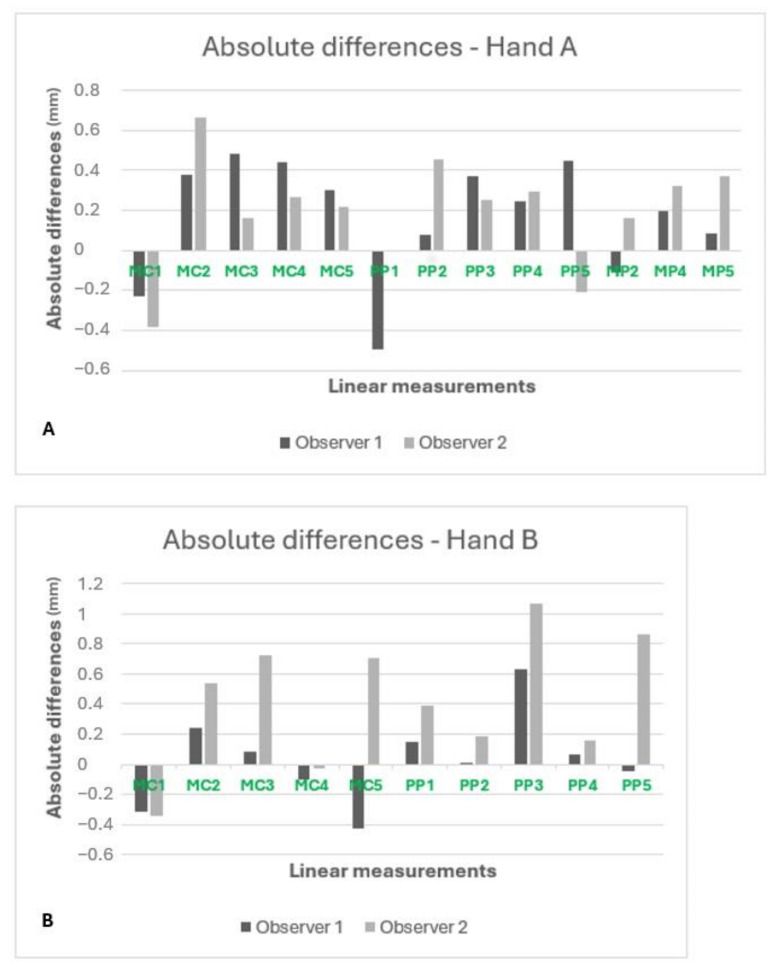
Mean absolute differences of linear measurements of each landmark for hand A (**A**) and hand B (**B**) performed by both observers. Distances measured by observer n°2 tended to be longer than measurements carried out by observer n°1. MC: Metacarpal; PP: Proximal phalange; MP: Middle phalange.

These values are accompanied by their respective absolute and relative errors. Both the mean absolute and dimensional errors were calculated for each hand and for the overall study. Two main facts were observed: the measured distances from the 3DP models were globally higher than on the cadaveric specimen, and measurements performed by observer n°2 tended to be higher in comparison to the other observer. This can be easily observed in Figure A1, which displays the mean absolute difference in each linear measurement in hands A and B, respectively, for both observers. Nevertheless, the level of errors and inaccuracies detected remained low. Indeed, for the overall study, the MAD was 0.32 mm (±0.34 mm) and the MDE was 1.03% (±0.83%). Therefore, our FDM AM process accuracy was good and below the recommended cut-offs of 1 mm for the MAD and 2% for the MDE, respectively.

Table 6 shows the *p*-values obtained by comparing the mean measurements from the cadaveric hands with their respective 3DP models. The measurement differences between hand n°2 and its 3DP replica appeared to be statistically significant. In order to understand that result, a second paired t-test was run, comparing the mean measurements from each observer, the corresponding *p*-values are gathered in Table 7. Only the comparisons between linear distances measured by observer n°2 were statistically significant. This seemed to follow the aforementioned observation that measurements taken by observed n°2 tended to be slightly higher than those of the first observer.

## 4. Discussion

As 3D printing becomes part of the clinical routine in healthcare facilities, ensuring the dimensional accuracy and reliability of AM processes should be incorporated in a quality management workflow [8,9].

In this study, we validated the dimensional accuracy and reliability of a hospital-based professional FDM 3D printer, finding an MAD of 0.32 mm (±0.34) and an MDE of 1.03% (±0.83). These values are below the recommended cut-offs and fall within the range of similar studies reporting the accuracy of FDM 3D printers and other AM processes (e.g., SLA, MJ, BJ) [8,13,31]. Table 8 summarizes the dimensional errors of comparable studies where the mean absolute and mean relative deviations vary from −0.055 mm to 0.65 mm and 0.08% to 3.76%, respectively; our models demonstrated comparable results [10,11,31,32,33,34,35,36,37,38].

Numerous works have investigated the dimensional accuracy of entry-level FDM 3D printers alone or compared to other 3D printing technologies [31,34,38]. Entry-level 3D printer advantages are affordability, accessibility and less complex management.

However, often these machines are limited in the type of different materials that can be printed and in their capacity for combining materials [1]. Professional FDM 3D printers, not necessarily unaffordable, allow the 3D printing of basic filaments (e.g., acrylonitrile butadiene styrene (ABS), polylactic acid (PLA)) but also of material with advanced mechanical properties (e.g., carbon, nylon, high-impact polystyrene (HIPS), polyether ether ketone (PEEK), etc.) [1,2,11]. Moreover, these printers might possess a dual nozzle capacity, allowing the combination of materials (or colors) [1]. The resolution can also be finer in professional FDM machines [1,2,11]. Therefore, in order to fully exploit the design space and the potential of the applicability of FDM printers, researchers, clinicians and engineers/designers should work together to determine the applications. This type of AM machine can be useful in, but not limited to, teaching or university hospitals.

Generally, other AM technologies have a higher resolution than FDM. However, FDM printers should not be considered inferior. Indeed, studies reported results showing a high level of accuracy for this technology, and sometimes being evenly or more accurate compared to other AM processes, especially Stereolithography [11,38,39].

For many applications, FDM is a suitable AM process. Moreover, the possibility to 3D print multi-color and/or multi-material models is a valid counterpart to a potential limited access to other 3D printing technologies [1,11] Furthermore, the final clinical application of 3D-printed specimens should always guide choices relating to the imaging data source, AM process and parameters (e.g., resolution), and material, as those decisions will impact the cost, accessibility and production time of the medical 3D-printed models of interest [8,11,38].

To date, there is no methodological gold standard to validate the accuracy of 3D-printed models from FDM professional AM printers [8]. Different approaches are reported in the literature: various comparison elements (cadaveric specimen, virtual 3D reconstruction, 3D models), different imaging modalities and radiological protocols, and numerous printing parameters [8,9]. The ways to perform accuracy measurements also varied: the number of observers and landmarks and their types, different measuring instruments and the number of measurement repetitions [8,14,31]. Therefore, it is crucial to determine a clear and accessible methodology to assess clinically 3D-printed specimens [6,14]. Some authors, such as Leng et al., attempted this task by developing a systematic approach: a “quality assurance” program that was more versatile than only taking measurements [9]. This paradigm ensures the reduction in errors in stages preceding the obtention of the 3D-printed models [9]. However, the absence of a gold standard hinders any comparison between accuracy assessment studies [6,31]. Moreover, it is essential for researchers to report technical parameters transparently, for the imaging and 3D printing stages to facilitate protocol comparisons.

Asaumi et al., suggested a cut-off at 2% to tolerate dimensional changes acceptable for surgical applications [13]. This value became a reference threshold for numerous works [1,9,31,39]. However, some authors have discussed its relevance, as the final applications might not systematically require a high level of accuracy [14,31]. Clinicians and operators must evaluate the necessity of precise replicas as their manufacturing can induce expensive costs and excessive building time, unsuitable for clinical routine [1]. For example, in their review, Mitsouras et al., indicated that relative differences up to 3% could be considered clinically negligible [1,9]. Moreover, the level of accuracy reported in this study could be considered high for the 3D printing layer height value chosen—300 µm. Indeed, this is considered as a low-resolution value in FDM technology, which reinforces the necessity to balance final applications with technical parameters [2,9]. In short, lower layer height values (i.e., higher *z*-axis resolution) do not automatically lead to a dramatic increase in dimensional errors [11,34].

The results of this paper are encouraging as they underscore that accessible FDM technology and a low resolution (300 µm) can already allow an accurate representation of hand bone anatomy. Furthermore, our results support the scarce evidence and reports of clinical applications of 3D printing to manage pathological hand conditions, such as in hand surgery (e.g., surgical training, pre-operative planning) and patient education [6]. Our data demonstrate the feasibility of the production of reliable anatomical replicas of human hand structures in a cost-effective manner. Therefore, hand surgeons, occupational therapists and other healthcare professionals managing hand health conditions should explore the added value of 3D printing in their respective clinical settings. Additionally, our results highlight that using alternative sources of radiological images, such as a CBCT scanner, still provides accurate 3D-printed medical models. Indeed, our study aligns with previous works investigating the usage of CBCT for limbs and musculoskeletal conditions [25,26,27]. Our paper underlines that CBCT can be used to both conduct radiological investigations and provide accurate digital reconstruction of hand anatomy and structures.

### 4.1. Possible Causes of Errors

Multiple factors impact the accuracy of 3D-printed models, and deviations can appear at any step of the manufacturing process. The critical stages are image acquisition, segmentation, printing and measurements [8,9,40].

CBCT was chosen as the imaging data source in this study as it constitutes a more accessible and affordable alternative to CT-scanner with the advantages of low radiation and faster acquisition time [25,41]. Moreover, some studies comparing both techniques, with CT-scanner as the gold standard, for the investigation of musculoskeletal conditions of extremities have demonstrated an equivalent, or higher, spatial resolution of CBCT with high bone contrast [25,27,42]. However, the importance of metal artifacts in CBCT acquisition in comparison to CT-scanner is not clearly defined in the literature [25,28]. Studies have validated 3D-printed models derived from CBCT for dental and maxillofacial applications [31,41,43]. However, to the best of our knowledge, none for the musculoskeletal applications for extremities were reported. A logical drawback of CBCT is its limited field of view (FOV) in comparison to the conventional CT-scanner, which can limit the size of the anatomical segments to be scanned [26,42]. In our study, the imaging data obtained from CBCT proved to be accurate.

The accuracy of the imaging data relies on a balanced trade-off of multiple essential parameters, such as the slice thickness, the slice spacing, the reconstruction Kernel and the signal-to-noise ratio, to name a few [7,9]. If not adequately set, these parameters would lead to poor imaging quality, causing inaccurate 3D reconstruction and 3D models. Slice thickness values have a direct impact on spatial resolution [1,7,8,9]. Indeed, acquiring thicker slice data can lead to reduced anatomic and tissue interface definition, as the signal from multiple tissues is averaged [1,9]. Therefore, to obtain optimal 3D reconstruction for 3D-printed models, a slice thickness below 1 mm should be prioritized [8]. Slice spacing represents another important factor as a high-distance factor can create gaps between slices, which could cause information loss in anatomical regions of interest [7]. Another parameter requiring a trade-off for better image resolution is the reconstruction Kernel (RK) algorithm, which impacts spatial resolution and image noise [7,9]. Consequently, the signal-to-noise ratio (SNR) also represents an essential factor to balance to acquire accurate radiological images [7,8,9]. Indeed, a high SNR and high contrast enhance the differentiation between structures on captured images and mitigate the partial volume effect [7,8]. Additionally, mitigating artifacts is also crucial as they can alter image quality. Therefore, established options such as electrocardiogram- or respiratory-gated acquisition should be considered when relevant and available [7].

These parameters influence the accuracy of imaging data, and thus the precision of computer modeling operations and the final model [7,8,9]. For instance, a poor trade-off between slice thickness and slice spacing can cause stair-step-like and discontinuous edges of the segmented digital model [7,9].

Likewise, unbalanced RK or SNR values can impede the precision of segmentation algorithms as structure delineation would lack sharpness [7,8,9]. Moreover, reconstruction and segmentation algorithms also possess limitations that impact their precision [8,9]. Indeed, both manual and automatic features, such as thresholding, region growing, filling or subtracting tools, can inadvertently modify models. Indeed, the unanticipated smoothing and wrapping of external surfaces, or the elimination of thin structures from original models, could occur during computation [8]. Thus, the final model might not reflect the initial source data.

**Figure 4:**
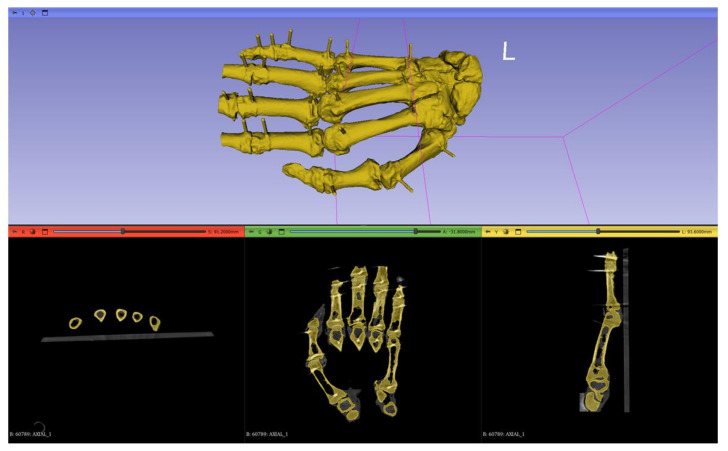
Segmentation phase of 3D digital hand model of hand A. Illustration of adequate overlaying of final 3D digal model on original imaging data in three views (axial, coronal, sagittal). “L” refers to the left side of the reference positioning cube, delineated by the pink lines, in the 3D view of the 3D slicer software.

The segmentation process can also be impacted by errors [8,9]. Specific software features such as threshold selection and the smoothing function can alter the quality of 3D-printed models [8,9,10,32]. For example, in our case, the ‘Closing holes’ feature of the smoothing function was necessary at times. Although on a gross inspection, this did not seem to affect the overall model anatomy, it may have caused minimal deviations. Moreover, manual segmentation was periodically required, which could also give room for small deviations. However, Leng et al., recommended a verification of the quality of the segmentation phase by overlaying 3D digital models on their original imaging data and inspecting the entire image set in all three planes (axial, coronal and sagittal) [9]. Figure 4 illustrates an example of relatively adequate overlaying between the imaging data of hand A of our study and its final digital model prior to its export as an STL file: osseous structures are correctly indicated as a Region of Interest (ROI) in color yellow, and soft tissues and forearm bones are excluded. Although the segmentation was considered accurate, small deviations could still have occurred, but they were negligible [7].

Measurements can also be prone to errors and impact statistical analysis [8,9]. This can be due to the systematic inaccuracies of measuring tools and human factors [14,32,39]. As multiple measurement protocols are present in the literature, determining an adequate evaluation is complex [8,14,39], especially as linear distances were measured manually in our study.

Therefore, in order to reduce the measurement error, several measurements were performed: twenty repetitions for each linear distance on each cadaveric hand and its 3D-printed replica. The measurement error induced by a human factor is often caused by the actual search of landmark points [10,14,32]. Therefore, the defined measurement landmarks and methodology must be precise and unambiguous, which was established in this study [8,14]. However, measurements values were influenced by the observers’ arbitrary closure level of the caliper, as the instrument precision was 0.01 mm. However, this manual method was demonstrated to be valid and reliable [7]. Additionally, the titanium wires inserted in the same bone segment were theoretically parallel to one another, as a guide was used. However, that condition may have been modified during the printing process, which would influence the measurements, as distances will vary along the height of two landmarks. Nevertheless, based on our results, we consider that situation negligeable.

The additive manufacturing process itself can be a source of error [8,9,39]. Indeed, modifications in model geometry or landmarks can appear due to material transformation, the layer deposition process or even the removal of support material [14,39]. Volumetric studies should be undertaken in order to assess such impacts on 3D-printed models.

**Figure 5:**
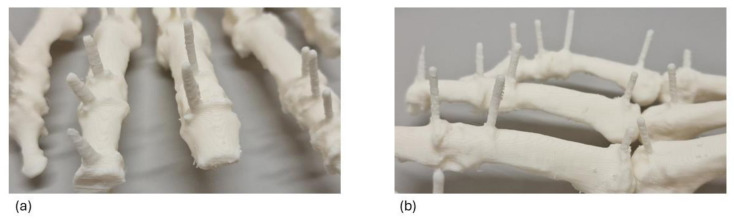
Unsmooth surface finish of 3D-printed model of hand A. (**a**) Dorsal view. (**b**) Lateral view.

In their princeps work validating the accuracy of a low-cost FDM 3D printer, Maschio et al., reported the difference in shape of some dental landmarks between the dry mandibles and their 3D-printed replicas [31]. Likewise, we could also observe physical modifications in our 3D-printed landmarks. Some of these could be explained by the angulation between the model on the build plate and the extruder, others by the position of original titanium wires at the distal extremity of the hand model that was at the edge of the scanning window. Differences in shape can also be due to the layer deposition process itself, which can cause an unsmoothed surface, as illustrated in Figure 5 [16]. However, despite these occasional incidents, the MAD was still within the acceptable range [16]. Such situations can be hindered by carefully determining the specimen position in the imaging modality apparatus and by fine-tuning the printing parameters in the slicer software [9].

Although some differences between the cadaveric specimens and 3D-printed models were statistically significant, all these differences were small and can be considered clinically negligeable. Other studies reported similar phenomenon [8,11,14,16].

The results of this study do not objectify the component of each error on the deviations of the 3D-printed models [14,35].

### 4.2. Limitations

This study also presents some limitations. Firstly, although a relevant number of landmarks were marked on the hand bones, not all phalanges were marked due to the technical limits of the CBCT modality. Therefore, further studies should investigate whether major variations in the MAD and MDE occur for a complete hand anatomy model, especially when including measurements of small structures such as distal phalanges. Additionally, landmarks were inserted only on the dorsum of the cadaveric hands. Although this approach demonstrated reliable accuracy, future works should consider adding landmarks on palmar faces of hand specimens and explore if a variation in precision occurs. Concerning the usage of CBCT, although its application for upper extremity disorders is promising and the calculated accuracy was within range, additional studies should compare these results with 3DP models derived from gold-standard imaging modalities (e.g., Multislice CT-scanner or Magnetic Resonance Imaging) in order to determine the most accurate method. Also, in this study, the set layer height was 300 µm, which corresponds to a common low resolution on entry and mid-level 3D printing. Further investigations should be undertaken to determine the relevance of opting for a higher resolution (i.e., a layer height inferior to 300 µm) in the production of hand models in most clinical contexts. Similarly, the only technology employed in this study, Fused Deposition Modeling (FDM), is currently the most common and affordable one. However, numerous studies have validated the accuracy, in other medical fields, of additional 3D printing processes such as Stereolithography or Selective Laser Sintering. These should be investigated for their application in hand care management. Finally, we did not scan the 3D-printed hand models with the CBCT scanner to acquire their 3D digital images. A comparison of the latter with the 3D images of the cadaveric hands, through superimposition, could have provided supplementary information concerning qualitative and quantitative errors in the methods. Errors induced at each processing stage (i.e., software, material, printing process) could hence be further explored.

In summary, despite the limitations mentioned above, our results support the validation of a hospital-based FDM 3D printing process. Further studies are desirable to establish guidelines for the production and accuracy assessment of 3D-printed models for medical purposes. Moreover, the usage of alternative imaging modalities, such as CBCT, should be investigated.

## 5. Conclusions

Our results showed that hospital-based FDM 3D printers have the potential to produce medical anatomical models with reliable accuracy. Moreover, CBCT appeared as a valid alternative to common imaging modalities (i.e., CT-scanner, MRI). However, validated guidelines are required for the development of robust institutional quality assurance workflows as healthcare facilities progressively implement 3D printing technologies in their care management. These quality control systems are crucial, as errors are susceptible to influence any stage of production of 3D-printed replicas.

## Figures and Tables

**Table 1:** Landmarks and linear measurements.

Landmark	Name	Measurement Definition
MC1	1st Metacarpal	Distance between base and head of metacarpal
MC2	2nd Metacarpal	
MC3	3rd Metacarpal	
MC4	4th Metacarpal	
MC5	5th Metacarpal	
PP1	1st Proximal phalange	Distance between base and head of proximal phalange
PP2	2nd Proximal phalange	
PP3	3rd Proximal phalange	
PP4	4th Proximal phalange	
PP5	5th Proximal phalange	
MP2	2nd Middle phalange of index	Distance between base and head of middle phalange
MP4	4th Middle phalange of ring finger	
MP5	5th Middle phalange of little finger	

MC: Metacarpal; PP: Proximal phalange; MP: Middle phalange.

**Table 2:** Printing characteristics.

	Printing Time	Weight (Grams)	Estimated Filament Cost (USD)
Hand A	9 h 27 m	98.5	2.5
Hand B	11 h 08 m	108.8	2.7

**Table 3:** Inter-observer agreement—ICC values.

	Hand A		Hand B	
Cadaveric	0.998	CI 95%: [0.987; 1.000]	1.000	CI 95%: [1.000; 1.000]
3DP Model	1.000	CI 95%: [0.999; 1.000]	1.000	CI 95%: [1.000; 1.000]

CI: Confidence interval; ICC: Intraclass Correlation Coefficient.

**Table 4:** Intra-observer agreement—ICC values.

Observer 1		Observer 2	
**Hand A**		**Hand A**	
1.000	CI 95%: [0.999; 1.000]	0.998	CI 95%: [0.981; 1.000]
**Hand B**		**Hand B**	
1.000	CI 95%: [0.999; 1.000]	1.000	CI 95%: [0.999; 1.000]

CI: Confidence interval; ICC: Intraclass Correlation Coefficient.

**Table 5:** Absolute difference, dimensional error, mean absolute difference (MAD) (mm) and mean dimensional error (MDE) (%).

Hand A—Cadaveric vs. 3DP Model				
Observer 1				
Landmark	Cadaveric (mm)	3DP Model (mm)	Absolute difference (mm)	Dimensional error (%)
MC1	35.20	34.89	−0.31	0.89
MC2	43.70	43.94	0.24	0.55
MC3	43.30	43.39	0.08	0.20
MC4	44.04	43.93	−0.10	0.23
MC5	43.66	43.23	−0.43	0.98
PP1	26.85	26.99	0.15	0.55
PP2	26.72	26.73	0.01	0.04
PP3	35.31	35.94	0.63	1.78
PP4	35.75	35.82	0.07	0.19
PP5	27.09	27.04	−0.05	0.18
		Average	0.21 mm (±0.20)	0.56% (±0.53)
Observer 2				
Landmark	Cadaveric (mm)	3DP Model (mm)	Absolute difference (mm)	Dimensional error (%)
MC1	35.18	34.83	−0.35	0.98
MC2	43.59	44.13	0.53	1.23
MC3	42.24	42.96	0.72	1.710
MC4	43.75	43.72	−0.02	0.06
MC5	42.40	43.10	0.70	1.65
PP1	26.75	27.14	0.39	1.44
PP2	26.62	26.81	0.18	0.69
PP3	35.31	36.38	1.06	3.01
PP4	35.61	35.76	0.16	0.44
PP5	26.30	27.16	0.86	3.28
		Average	0.50 mm (±0.34)	1.45% (±1.03)
**Hand B—Cadaveric vs. 3DP Model**				
Observer 1				
Landmark	Cadaveric (mm)	3DP Model (mm)	Absolute difference (mm)	Dimensional error (%)
MC1	35.19	34.96	−0.23	0.65
MC2	51.97	52.35	0.38	0.73
MC3	52.31	52.79	0.48	0.93
MC4	43.79	44.23	0.44	1.01
MC5	43.59	43.89	0.30	0.69
PP1	18.36	17.87	−0.50	2.71
PP2	35.44	35.52	0.08	0.23
PP3	35.23	35.60	0.37	1.06
PP4	35.66	35.90	0.24	0.68
PP5	26.44	26.89	0.45	1.70
MP2	10.42	10.31	−0.11	1.06
MP4	19.03	19.23	0.194	1.02
MP5	10.37	10.45	0.08	0.81
		Average	0.30 mm (±0.15)	1.02% (±0.61)
Observer 2				
Landmark	Cadaveric (mm)	3DP Model (mm)	Absolute difference (mm)	Dimensional error (%)
MC1	35.17	34.79	−0.38	1.09
MC2	52.17	52.83	0.66	1.27
MC3	52.58	52.74	0.16	0.31
MC4	43.75	44.01	0.27	0.61
MC5	43.71	43.93	0.22	0.50
PP1	17.74	17.73	−0.01	0.04
PP2	35.50	35.96	0.46	1.29
PP3	35.31	35.56	0.25	0.71
PP4	35.62	35.92	0.29	0.83
PP5	26.76	26.55	−0.21	0.77
MP2	10.11	10.28	0.16	1.62
MP4	18.83	19.15	0.32	1.70
MP5	10.08	10.45	0.37	3.70
		Average	0.29 mm (±0.16)	1.11% (±0.92)
				
		Global MAD	0.32 mm (SD: 0.34)	
		Global MDE	1.03% (SD: 0.83)	

3DP Model: 3D-printed model.

**Table 6:** Paired samples *t*-test results for global study.

Cadaver—3DP Model	*p*-Value	Significance *
Hand A	0.05	No
Hand B	0.02	Yes

*: statistically significant at *p* < 0.05.

**Table 7:** Paired samples t-test results by observer.

Observer	Hand	*p*-Value	Significance *
1	1	0.77	No
1	2	0.06	No
2	1	0.01	Yes
2	2	0.02	Yes

*: statistically significant at *p* < 0.05.

**Table 8:** Overview of comparable studies assessing the accuracy of 3D-printed models.

Study	Anatomical Specimen	Imaging Technology	CT Slice Thickness	Segmentation Software	3D Printing Technology	Difference (Absolute)	Difference (Relative)
Our study results	Hand	CBCT	0.25 mm	3D-Slicer version 5.61. (Brigham and Women’s Hospital Inc., Boston, MA, USA)	FDM	0.32 mm (±0.34)	1.03% (±0.83)
Choi et al., (2002) [32]	Skull	CT-Scanner	1.0 mm	V-Works (Cybermed Inc., Seoul, Korea)	SLA	0.62 mm (±0.53 mm)	0.56% (±0.39%)
Nizam et al., (2006) [10]	Skull	CT-Scanner	1.25 mm	Mimics (Materialise NV, Leuven, Belgium)	SLA	0.23 mm (±1.37 mm)	0.08% (±1.25%)
El-Katany et al., (2010) [37]	Skull, Mandible	CT-Scanner	N/A	Stratasys (Stratasys, Eden Prairie, MN, USA)	FDM	Mandible: 0.079 mm (±0.031) Skull: 0.108 mm (±0.048)	Mandible: 0.22% (±0.11) Skull: 0.24% (±0.16)
Petropolis et al., (2015) [34]	Dry skull Mandible	CT-Scanner	1 mm	Osirix (Pixmeo, Geneva, Switzerland)	FDM SLS	FDM: 100 µm: 0.21 mm 250 µm: 0.24 mm 500 µm: 0.56 mm SLS: 0.16 mm	100 µm: 0.44% 250 µm: 0.53% 500 µm: 1.1% SLS: 0.30%
Maschio et al., (2016) [31]	Mandible	CBCT	0.5 mm	Maxilim (Medicim, Mechelen, Belgium)	FDM	0.37 mm	3.76%
Rendón-Medina et al., (2018) [33]	Mandible	CT-Scanner	1 mm	3D-Slicer (Brigham and Women’s Hospital Inc., Boston, MA, USA)	FDM	0.65 mm	1.96%
Reddy et al., (2018) [36]	Lower limb bones (femur, tibia, talus, …)	CT-Scanner	0.625 mm	InVesalius (Centro de Tecnologia da Informação Renato Archer, Campinas, SP, Brazil)	FDM	0.40 mm	N/A
Msallem et al., (2020) [11]	Mandible	3D-Scanner	N/A	N/A	SLS SLA MJ BJ FDM	SLS: 0.11 mm (±0.016) SLA: 0.45 mm (±0.044) MJ: 0.21 mm (±0.02) BJ: 0.14 mm (±0.02) FDM: 0.16 mm (±0.009)	N/A
Hatz et al., (2020) [38]	Mandible	CT-Scanner 3D-Scanner	N/A	Materialise 3-matic (Materialise NV, Leuven, Belgium)	FDM SLS	FDM: −0.055 mm (±0.227) SLS: −0.019 mm (±0219)	N/A
Kaschwich et al., (2021) [14]	Abdominal aorta	CT-Scanner	1.000	Mimics (Materialise NV, Leuven, Belgium)	Poly-Jet	−0.73 mm to 0.14 mm	−2.78% to 1.71%
Ravi et al., (2022) [35]	Multiple organs/pathologies (Kidney, Mandible, Glioma, aneurysm, etc.)	CT-Scanner	0.625 mm	N/A	FDM	0.26 mm (±0.14 mm)	0.71% (±0.33%)

BJ: Binder Jetting; CBCT: Cone Beam Computed Tomography; CT: Computed Tomography; CT-scanner: Computed Tomography scanner; FDM: Fused Deposition Modeling; MJ: Material Jetting; N/A: Not applicable; SLA: Stereolithography; SLS: Selective Laser Sintering.

## Data Availability

The data used to support the findings of this study are available from the corresponding author upon request.
